# After the pandemic is before the pandemic: Rethinking urban priorities, assumptions and planning approaches

**DOI:** 10.1016/j.heliyon.2023.e20763

**Published:** 2023-10-06

**Authors:** Xincheng Zhu, Jiaxiang Xu

**Affiliations:** a School of Planning, University College London, London, WC1H 0QB, UK; b Institute of Archaeology, Faculty of Social & Historical Sciences, University College London, London, WC1H 0QB, UK

**Keywords:** Urban planning, Covid-19, Challenges, Post-pandemic

## Abstract

The global covid-pandemic had significant impacts upon cities and city planning in the short, medium and longer term. Early severe disruptions to city living and working patterns prompted interventions from planners and the suspension of plans, largely based on projections of pre-pandemic data and trends. Longer term thinking has begun to settle into a pattern of reactions and approaches depending upon the characteristics of the city. This paper explores some of the responses to the pandemic and how cities are adapting and evolve towards a ‘new normal’ and what this might mean for planning and planners in future. Two questions are posed for future research following from this. First, will planners require new skills and knowledge going forward as cities adapt and evolve and, second, how can we better comprehend the full spectrum of responses and trajectories of city planning post-pandemic.

## 1 Introduction

Since early 2020 the world's attention has been focused on the Covid pandemic. Whilst a global phenomenon, it is cities that felt the brunt of the pandemic's impact [1]. The reasons for this are twofold. First, as is commonly pointed out, we are an urban species with more than half the world's population living in cities and this proportion projected to rise to 68 % by 2050 [2]. Second, Covid is a contagious disease transmitted mainly through breathing in contaminated air particles. Physical distance reduces the likelihood of transmission which is more difficult to achieve in cities given the number of people and propinquity of urban living and working. This confluence of large, urban populations often living at high densities combined with an airborne disease has meant that the pandemic has disrupted the city in a wide range of ways [3,4]. Four major changes stand out.

First, the relationship between the pathogen and the city led many to seek to relocate beyond the urban to less dense, commuter towns in order to reduce the likelihood of infection, a reaction to epidemics that is has been repeated throughout history. The necessity for many of home working and schooling also created a demand for more and different kinds of space further fuelling a population shift, increasing property and rental values in suburban areas and reducing them in cities [5]. However, this flight from the city was confined to those lucky enough to have the opportunity or means to relocate and/or work from home. As ever, it was the urban poor that were hardest hit by Covid including those who had little choice but to continue to work and live in the city. This group included the estimated 1 billion people around the globe who live in overcrowded, informal settlements and have variable and limited access to clean air and water, sanitation, and power not to mention a reduced ability to physically distance.

Alongside significant population movements a second major related pandemic shift has been the accelerated move to online working and online retailing [6]. During the pandemic footfall in cities plummeted and whilst there has been some recovery many cities are facing an uncertain future as shopping and leisure habits shift [7,8]. The shift online was underway pre-covid though was accelerated greatly as a result of the pandemic raising questions for some over the longer term impacts and resilience of places that are particularly dependent upon, for example, tourism and hospitality [9]. A related third major change has been the impact of the pandemic upon public infrastructure needs, specifically public transport. Early in the pandemic public transport was presented and perceived as a vector of virus transmission [10], encouraging more active forms of travel such as walking and cycling as well as a modal shift back to private transport use. The overall demand for public transport fell dramatically though some return to close to pre-pandemic levels has been experienced in most countries. Nevertheless, longer term plans and investment in future public transport infrastructure were, at the very least, now open to question. Fourthly, there has been widespread economic disruption to cities which have varied between sectors and places, underlining existing inequalities whilst exacerbating others. Finally, demand for existing spaces shifted as people sought out new places and spaces that provided more opportunities for social and physical distancing, challenging existing patterns of leisure, cultural and tourism. Use of open, urban spaces, such as parks, increased whilst local municipalities sought to repurpose existing spaces to provide more opportunities for physical and social distancing [11].

As the vaccination programme began to change the pandemic from a crisis to an on-going challenge in many countries the longer-term impacts and implications for cities and city planning continued to be played out. In a process and profession that values certainty there was and remains considerable uncertainty over how the pandemic will change attitudes to, for example, locational preferences for employers and employees and the consequences for public transport use, housing demand and supply, leisure and recreational use and the need to refocus upon health as a driver of city planning [12]. This paper explores some of the responses to the pandemic and how cities are adapting and evolve towards a ‘new normal’. The broad experience is that planners and city authorities are ‘feeling their way’, developing responses and approaches that reflect local circumstances and amount to a global ‘experiment’ in post-pandemic urban policy [13]. Some cities are also using the pandemic as an opportunity to introduce changes that would in other times be controversial and contested. After charting some responses and trajectories of change we then draw some wider conclusions and observations on how cities and city planning are facing up to the challenges of the pandemic and what this might imply for future urban policy.

## 2 The challenges to cities and urban planning

Modern city planning can trace its origins back to concerns over health related outbreaks of plague, cholera and yellow fever across Europe. Iconic urban redevelopments like Hausmann's boulevards of Paris were created, in part, by the need to improve sanitation. In the UK, the first Housing Act in 1890 emerged as a reaction to the poor health standards across Victorian slums whilst land-use planning and zoning systems emerged as by-products of the need to improve sanitation and health, particularly following the Spanish influenza outbreak of the years 1920s across Europe. Yet as conditions improved as a result of such interventions health related urban planning shifted in many developed countries to focus on non-communicable, so called life-style diseases such as obesity whilst in developing countries infectious diseases remained the priority [14].

Given this shift away from infectious disease as a foundational purpose for urban planning there was a lack of knowledge and skills from within the sector when faced with the sudden need to respond to a pandemic in 2020. For many if not most who were involved in city planning this was a new and unfamiliar place, one that was disruptive but without a clarity on ways forward or necessary actions [15]. Strategies and plans based on pre-pandemic data and forecasts and long-standing, professional methodologies were replaced by the need for rapid reaction, often based on uncertainty and incomplete information. The World Health Organisation provided a direction of travel for cities in the face of such disruption through its advocacy of the ‘3 Cs’: avoiding crowded spaces, close contact and confined places. City authorities around the globe followed this approach to greater or lesser degrees, helping facilitate remote working and active travel, challenging many long-held assumptions and models of cities [16], shifting political attention away from existing policy frameworks around climate change and sustainability [17].

This period of immediate response was soon accompanied by wider debates on the future of cities. Given the disruptive shifts as large parts of society moved online and out of cities combined with the uncertainty over infection and fatality rates there was speculation on whether cities were facing an existential challenge (for example, [18]. Remote working and online retailing had been growing prior to the pandemic but with an estimated 40 % of jobs being capable of being undertaken wholly and largely remotely the prospect of a permanent shift in working patterns would fundamentally challenge cities. A permanent shift towards greater online working, even if partial, would reduce the demand for public transport, services and retail as well as office and residential space in cities [19,20]. Related consequences of such a change would be on longer term policy positions and trajectories around the aim of addressing climate change through increasing development densities and investing in public transport. Commercial considerations also began to feed into the questioning of the city as some firms began to embrace the idea of flexible working as a means of reducing rents and outgoings. Serious questions were asked around whether or not urbanisation had peaked and the future for cities would look very different.

Whilst the disruption caused by the pandemic was immediate and serious it was eclipsing some longer term, serious urban challenges:

Contagious disease is the most obvious threat to urban life in 2020, but it is not the only one. A pandora's box of urban woes has emerged including overly expensive housing, violent conflict over gentrification, persistently low levels of upward mobility.

The flight from the city might have been initiated by the pandemic but it reflected deeper trends and issues.

Yet a different perspective argued that what was happening in cities wasn't an existential moment but more of a disruption, a familiar and regular occurrence that cities have faced throughout their history [21]. According to this view cities had been disrupted by technological innovations such as the internal combustion engine, by environmental challenges such as extreme weather events, conflicts and wars as well as economic recessions. The global covid pandemic was framed by some commentators as akin to such historic disruptions which would ultimately lead to adaptation and evolution of the city. New design criteria that included space within and around buildings in order to increase ventilation and physical distancing were proposed whilst an important distinction between high density and overcrowding was made to counter the claim that a post-pandemic city was incompatible with a sustainable city (for example, [22].

A related stream of thought on the impact on cities also highlighted what some saw as the positive changes wrought by the pandemic. Grounded planes and dramatic reductions in car-based transport led to significant falls in carbon emissions whilst the reduction in demand for city living led to a fall in rents and an improvement in housing affordability. Others highlighted how some of the changes in work patterns forced by the pandemic accelerated the move towards what has been termed the ‘new urban economy’, with part-time, flexible working reducing congestion and allowing people to live more sustainable lives [16]. Such optimism overlooked the fact that much of what had been written and opined on the future of the city based on digital enabling ignored the many who did not have the option of working from home, those charged with providing critical functions including health services, water and sanitation provision, and the delivery of food and other necessary items. Working from home for some was heavily dependent on many not working from home and keeping essential goods and services available (see Ref. [23].

## 3 The responses from cities and urban planning

The period from the beginning of 2020 to towards the end of 2022 was an era of significant uncertainty, pragmatism and experimentation for cities and urban planning [24]. As cities around the world began their journeys back to some form of normality-however that looked-patterns began to emerge in common actions and debates across a range of cities that sought to address some of the impacts of the pandemic. These actions varied though three broad categories of change can be identified.

### 3.1 Rethinking priorities

Cities and city planning juggle a range of multiple, dynamic, complex and sometimes conflicting demands and challenges. Such challenges, include housing affordability, inequality, crime, air pollution and traffic congestion, to name a few. On top of these localised factors sit more global challenges including climate change and the disruption of digital technology [25]. Overarching policy approaches such as the drive towards sustainable development provide policy direction and options but do not always overcome the need to reconcile competing options, e.g., environmental protection versus economic growth. Neither do they help overcome some of the tensions and concerns inherent in some policy options, particularly the unease over privacy concerns over the continued roll-out of smart city infrastructure, a roll-out that was accelerated and deepened during the pandemic on the justification of health related necessity (see, for example, [[26], [27], [28], [29]]. The pandemic has added a further dimension to this through the emphasis given to health and the need to plan for what is widely seen as the inevitability of another pandemic [30]. According to Gandy (2022) the pandemic has brought epidemiological questions into sharp focus and has added to the set of vulnerabilities that cities face and how institutions frame public health issues alongside other challenges and objectives, including climate change.

The calls for city planning to take on board preventative, adaptive and mitigating measures to help address future public health disruptions now sits firmly on the planning and city agenda [[31], [32], [33], [34], [35]]. But this agenda is already full and elements of it cannot easily be reconciled, e.g., should cities densify in order to help tackle climate change or seek to reduce densities in order to create space as part of the healthy cities agenda? There are some who reject this binary choice and, instead, see this as a common and mutually compatible journey (e.g., Refs. [[36], [37], [38]]; Searle and Turnbull, 2020). As well as potentially irreconcilable issues this full agenda raises another set of questions for the post-pandemic city, including how will a focus on health and questions over city-strategies take attention away from other challenges that cities face, e.g., digital technology, climate change, inclusion, housing affordability, economic growth and the need to tackle inequality?

One way in what this more crowded list of priorities and objectives for cities and city planning has been managed was through a general invocation that the sacrifices that individuals were making through lockdowns, etc. Would be worthwhile: the UK's rallying call to Build Back Better (and the variations of this soundbite around the globe) sought to paint a picture of progress and improvement; of gain after the pain. Little detail was provided as to how ‘better’ might feel or look as, understandably, there was a lack of certainty around the full impact and longevity of the pandemic. This hope and vagueness helped provide the certainty and reassurance that some felt necessary, particularly regarding public and private investment - cities would endure and that the fundamental elements of what made them necessary and successful would remain [15]. There was necessary reassurance that historic and future public and private city investments would be ‘safe’ in order to ensure continued regeneration and redevelopment activity. Another way in which cities juggled on-going policy frameworks against the emergence of a new and largely uncertain challenge in the guise of the pandemic was to pause and rethink of priorities within city planning. For many places this involved reflection on how and to what extent existing policy priorities such as climate change could take on board the increased emphasis on health [39].

Reckien, for example, offered an early attempt to explore the synergies between the post-pandemic city and the climate emergency whilst Marshall suggested that because the impacts of the global pandemic were very personal and local then this raised the possibility that people will be more willing to act on other global challenges such as climate change. Pre-covid initiatives such as the ‘15 min city’ received a new lease of life through incorporating various strands that echoed elements of how cities were evolving to incorporate health into existing policy frameworks such as sustainability. The emphasis upon active travel, quality public space, remote working, mixed uses developments and accessible local services [40]. The overlaps and synergies between the emphasis within sustainable development upon climate change related health issues such as vehicle-related air pollution, temperature rises and weather extremes point to similar emphases upon policies to increase ‘micro-mobility, reduce travel distances to meet basic needs, increase the use of mass transit, change consumption patterns to reduce industrial production and strengthen the circular economy are necessary to manage increased CO_2_ and carbon emissions and improve air quality’.

There are two other related ways in which this new addition to the pantheon of policy considerations has impacted upon cities through rethinking the assumptions that underpin planning and the approach to planning itself.

### 3.2 Rethinking urban assumptions

City planning is founded on a range of underlying assumptions that in many place amounts to a ‘predict and provide’ model. Crudely, demographic change through migration, birth rates and life expectancy provide cities with a broad understanding of supply and demand that is then disaggregated into sectors such as employment, housing, transport, leisure, etc. Which can then be translated into spatial policies and land-use zoning. The Covid pandemic, at the very least, disrupted such underlying thinking and, as some claim, upended convention conceptions of the drivers of demand and supply in urban space [41].

Against this backdrop existing assumptions and models based on projecting past trends into the future have been seriously challenged [42]. Work by the Urban Land Institute, for example, highlighted the underlying assumptions of large, city centre-based employers on future ways of working. This globally focused research pointed to the working assumption of around 60 % of workers spending around 40 % of their time working from home in future, reducing the demand for city centre office space, public transport use and other, associated city-centre services and amenities such as bars, restaurants and leisure. The kinds of space that employers are looking for in this new context of demand has also changed with a shift towards space that facilitates collaboration and innovation in the time when employees are in the office.

At the same time demand for office, leisure and retail space in suburban and peripheral small town locations has increased as those working from home shift consumer patterns. One associated challenge with this is the need to reallocate urban space and revisit projects of supply and demand. The City of London is developing proposals to reallocate current and likely future empty office space for affordable housing [43]. In addition to the need to revisit assumptions around supply and demand there are two, further broad consequences of this. The first is the need for greater flexibility in zoning to allow for adaptation in uses and demand (Rethinking Planning below). The second is the focus these changes place on the need for more emphasis upon city promotion and marketing [44].

### 3.3 Rethinking planning

It is not only the underlying assumptions of city planning that have been impacted by the pandemic but the tools, processes, knowledge and skills of planners and planning too. Whilst city authorities around the globe reacted to the pandemic beyond the necessary and reactive short term needs [44] identifies a range of longer term responses including changes to zoning and land use allocations, interventions to improve liveability and shifts in approaches to mobility and accessibility (see also [[45], [46], [47]]. In terms of zoning and land use cities have had to take a more flexible attitude towards traditional city centre uses as demand for office, commercial, retail and leisure space has declined based on the assumption that it is unlikely to return to pre-pandemic levels. In London and many other UK cities policies to protect the conversion of office space to other uses has been relaxed, allowing more mixed use schemes including affordable housing to emerge. In some US cities this flexibility has extended to hotels as well as offices and has been accompanied by tax incentives to encourage the development of apartments in under-used, post-pandemic office space. Many cities have also begun to focus on how to encourage residents and workers back into cities through improvements to city centre environments whilst also recognising that the locational preferences of urban residents and users has shifted. As many no longer prioritised the close connection between home and work and, instead, gave greater primacy to parks and green/open spaces as well as other, pandemic-led ‘quality of life’ priorities such as museums, restaurants and schools. Finally, cities such as Milan, Auckland, Buenos Aires have initiated investments and interventions in active transport such as cycle lanes and footpaths along with widening pavements to create more space for restaurants, bars and cafes, pedestrianizing streets and closing off others to through traffic.

Overall, there has been a realignment of planning, shifting timescales from the long term to the shorter term through interventions to tackle the impacts of the pandemic [48]. Planning has also sought to fuse the re-emergence of health-related concerns within existing and on-going policy frameworks such as sustainability and revisit concepts such as resilience, previously linked to climate change but now more broadly conceived as including health related issues too. However, as a number of authors have pointed out, city planning has been forced to act and is more likely to have to continue to act on shorter timescales and more ‘tactically’ through strategic improvisation.

## 4 Conclusions

It is misleading to talk of a post-pandemic world never mind a post-pandemic city given that we have lived through a number of such outbreaks and we are warned that more will come: the influenza pandemic of 1918–1919, Asian Flu (1957–1958), HIV/AIDS (1980 onwards), H1N1 flu (2009), Ebola (2013–2020) and the Zika virus (2015–2016), to mention a few, highlight that pandemics are nothing new. Yet the outbreak of the current pandemic has led to some fundamental changes in our patterns of living and working, changes that will play out in our cities over a long period, will land differently in different places and will interact in sometimes unpredictable ways as they interact with on-going disruptions brought about through digital technology [49]. What is clear is that such disruptions are having impacts on the way we experience and plan our cities. For some such as Glaeser and Cutler the longer-term impacts will be: less of a transformation than many predict. While some cities are in danger, the downtown in most places is far from dying. A greater share of the routine and easily evaluated work will be done at home, probably saving one or two commutes per week, but the most important moments on the job will still happen around co-workers. Commercial rents will certainly fall, and some commercial space will be converted to residences or allocated to scrappier start-ups. But the city itself will continue to be a home to rich and poor.

It is also worth bearing in mind that not all of the impacts and consequences of the pandemic on cities are necessarily negative – there are opportunities to accelerate some policy agendas and progress new ideas and changes particularly around carbon reduction and air quality in cities. Such opportunities are diminishing, however, as we come out of the immediate ‘crisis’ phase of the pandemic and move back into more business as usual the visibility and immediacy of health related, pandemic-focused priorities diminishes. The significant, pre-existing and on-going challenges that cities face, from climate change to economic and social inequality, resurface and could well eclipse the lessons learned and the necessary shifts and changes required to prepare for the next pandemic.

Research and analysis around the post-covid city are on-going in a wide range of disciplines, not least urban planning and the complete picture will take some time to emerge. Two areas that will require further reflection come out of our review. The first is that there is clearly no dominant paradigm or consistent reaction to the pandemic. Not surprisingly, cities have adapted and evolved in different ways largely dependent upon a range of factors, including the individual circumstances and experiences of the pandemic. Learning from other places is a key element of urban policy and the first step in that is the need to develop greater understanding of the myriad of approaches-a typology of places and responses to help frame analysis and further research. Second, it was obvious from the early responses to the pandemic that planners and planning were inadequately equipped to deal with major disruptions to long standing trends and approaches. New skills and knowledge were quickly sought and more will be needed in future because, as we are constantly reminded, we have experienced many, similar outbreaks through the 20th and 21st centuries and we can expect another. So, preparation will require reflection on what kind of urban form and planning as well as methodologies planners should be adopting, including greater understanding of, for example, epidemiology. If cities are going to experience longer term shifts in working and living patterns then this might require new approaches to, say, urban renewal-what are the underlying demands and preferences that shape such necessities? These and other questions will be posed of planners and city planning around the globe.

## Author contribution statement

All authors listed have significantly contributed to the development and the writing of this article.

## Data availability

Data included in article/supp. Material/referenced in article.

## Additional information

No additional information is available for this paper.

## Declaration of competing interest

The authors declare that they have no known competing financial interests or personal relationships that could have appeared to influence the work reported in this paper.
